# Types of Insanity

**Published:** 1883-11

**Authors:** 


**Types of Insanity:** An Illustrated Guide, with Physical Diagnosis of Mental Disease. By ALLEN MCLANE HAMILTON, M. D., one of the physicians to the Insane Asylum of New York City and the Hudson River State Hospital for the Insane. New York: William Wood & Co., 56 an 58 Lafayette Place. 1883.

This work aims to present to the general practitioner, for whom it is especially written, in a simple form, "a few suggestions" that may prove useful in meeting cases of insanity in their practice. The various types of the disease are illustrated by plates drawn from instantaneous photographs, the subjects being selected from hundreds of patients and the cases typical. The author presents a clear introduction to the subject of insanity, and then devotes a chapter to the general appearance of the insane; a chapter on the condition of special organs, the eyes, the ears, the mouth, the teeth, the tongue, the nose, etc.; a chapter on the condition of the bodily functions, the circulation, the temperature and pulse variations, the skin and its appendages, muscular tonus, the reflexes, sensibility, the urinary secretion, menstruation; a chapter on the examination of patients, changes in dress and personal habits, the handwriting of the insane; a chapter on the commitment of the insane, with abstract of the laws of the various States.

The work contains plates illustrating typical cases of idiocy, imbecility, melancholia attonita, chronic melancholia, subacute mania, chronic mania, dementia, general paresis, affections of the ear and condition of the teeth in the insane.

It will be seen that the work supplies to the general practitioner a guide in the examination of insane patients, in the diagnosis of the various types of insanity, a guide which will be of great service now that insanity has become a frequent disease, for the exercise of his judgment and skill; and we think the enterprising publishers have not only chosen wisely in the selection of the author for the preparation of the work, but have succeeded admirably in presenting it in a form that will command the approval and patronage of the profession.

